# A Low-Complexity Compressed Sensing Reconstruction Method for Heart Signal Biometric Recognition

**DOI:** 10.3390/s19235330

**Published:** 2019-12-03

**Authors:** Jian Xiao, Fang Hu, Qiang Shao, Sizhuo Li

**Affiliations:** School of Electronic and Control Engineering, Chang’an University, Xi’an 710064, China; 2018232035@chd.edu.cn (F.H.); 2017232021@chd.edu.cn (Q.S.); 2019132045@chd.edu.cn (S.L.)

**Keywords:** compressed sensing, bioelectrical signals, signal reconstruction, biometrics, energy consumption optimization

## Abstract

Biometric systems allow recognition and verification of an individual through his or her physiological or behavioral characteristics. It is a growing field of research due to the increasing demand for secure and trustworthy authentication systems. Compressed sensing is a data compression acquisition method that has been proposed in recent years. The sampling and compression of data is completed synchronously, avoiding waste of resources and meeting the requirements of small size and limited power consumption of wearable portable devices. In this work, a compression reconstruction method based on compression sensing was studied using bioelectric signals, which aimed to increase the limited resources of portable remote bioelectric signal recognition equipment. Using electrocardiograms (ECGs) and photoplethysmograms (PPGs) of heart signals as research data, an improved segmented weak orthogonal matching pursuit (OMP) algorithm was developed to compress and reconstruct the signals. Finally, feature values were extracted from the reconstructed signals for identification and analysis. The accuracy of the proposed method and the practicability of compression sensing in cardiac signal identification were verified. Experiments showed that the reconstructed ECG and PPG signal recognition rates were 95.65% and 91.31%, respectively, and that the residual value was less than 0.05 mV, which indicates that the proposed method can be effectively used for two bioelectric signal compression reconstructions.

## 1. Introduction

In recent years, with the development of embedded and wireless network communication technologies, many wearable and portable cardiac signal acquisition devices have emerged, making it increasingly less difficult to detect signals. Cardiac signals have also been widely used in various fields, and the identification technology based on cardiac signals has become a research hot spot [1,2]. However, difficulty lies in combining the remote identification technology of heart signals with portable heart signal acquisition equipment to simultaneously improve the accuracy and convenience of identification and meet the requirements of identification systems regarding efficiency and accuracy. Compressed sensing (CS) is a new sampling theory that uses a fixed set of linear measurements together with a nonlinear recovery process. CS aims to reconstruct signals and images from significantly fewer measurements. The characteristic of compressed sensing [3,4] is that its sampling and data compression are synchronously completed, avoiding the collection of a large amount of redundant information, reducing the complexity of the data, and improving the compression performance of the data. The application of compressed sensing technology to portable cardiac signal identification devices can alleviate the problem of generating a large amount of redundant data during cardiac signal acquisition, thereby reducing the occupied storage space and energy consumption [5,6] of data transmission and improving the performance of portable devices.

Traditional strategies for identification rely on entities (tokens or ID cards) or passwords. However, such strategies are based on something that the user knows or possesses and are thus vulnerable to security attacks. Biometrics are characteristics extracted directly from human subjects to form signatures for identity verification systems. By designing an authentication key to be highly correlated to physiological or behavioral features of an individual’s identity, this class of strategies offers airtight security. Several biometric modalities have been used so far, among which are fingerprints, face, voice, keystroke, and gait. Although most of them have gained wide acceptance, the main limitation in their application is their defenselessness against falsification. Lately, attention has been given to employing a new biometric trait: the electrocardiogram (ECG). ECG signals reflect cardiac electrical activity and have been well studied for medical diagnostic purposes. The idea of identifying subjects by ECG is relatively new, but it has considerable advantages. The key benefit is the robustness against falsified credentials, as it is difficult to steal an ECG and impossible to mimic it. In addition, an ECG itself is a liveness indicator, which suggests that potential applications will need to ensure that the subject who is offering the biometric is indeed the one who is carrying it. This is not the case with conventional biometrics such as fingerprints, irises, and faces, which are deficient in situations where additional mechanisms are needed to guarantee liveness [7]. Human recognition based on ECG signals is a relatively new and rapidly developing method [8]. Compared with the human face, fingerprints, irises, and other biological features, heart telecommunication signals have the advantages of ensuring that there is a living body, ease of collection, difficulty of being stolen, and so forth, which is why they have gained an increasing amount of attention in the field of identification. 

In this work, we mainly studied a cardiac signal reconstruction method based on compressed sensing. We focused on ECGs and photoplethysmograms (PPGs) in cardiac signals. In the phase of signal reconstruction, the stagewise weak orthogonal matching pursuit (SWOMP) algorithm was improved to enhance the accuracy and stability of reconstruction. Finally, the eigenvalues of the reconstructed cardiac signals were extracted for identification, and the accuracy of the proposed method and the practicality of compressed sensing in cardiac signal identification were analyzed, providing support for cardiac signals for identification. In summary, we made the following major contributions:

- Research on a preprocessing method of ECG signals based on the wavelet transform algorithm of coif3 function. This method can effectively remove noise and provide a clean ECG signal for subsequent ECG signal processing with low computational complexity.
- Study of ECG and PPG signals feature extraction based on discrete wavelet transform. After denoising the signal by wavelet transform, the wave bands of different frequency segments are located by setting the corresponding threshold and the value window. Finally, each waveform is located. The algorithm can effectively extract the features of ECG and PPG signals.
- Research on ECG and PPG signals compression and reconstruction based on compressed sensing. The sparsity of ECG and PPG signals was studied, and an improved reconstruction algorithm was designed. The proposed method was used to compress and reconstruct the ECG and PPG signals, and the reconstruction time and data error under different conditions were obtained, providing support for practical application.
- With the standardized reconstruction error calculation method and signal reconstruction time as the evaluation index, the reconstruction algorithm was comprehensively analyzed. ECG data of the same lead were used to reduce the influence of uncertainty factors on the results.

This article is organized as follows. Section 2 introduces some of the most relevant related works that can be found in the literature. Section 3 mainly considers the compression reconstruction of two bioelectrical signals (ECG and PPG) using compression sensing technology, the design of the reconstruction algorithm, and an analysis of its performance. Section 4 introduces the application of compressed sensing in bioelectric signal recognition, the feature extraction method of two kinds of signals, and the classifier used for signal recognition. Section 5 describes the simulation experiments performed to verify the feasibility of the proposed method. Finally, Section 6 summarizes the paper and proposes future work directions.

## 2. Related Work

T. Tao, D.L. Donoho et al. [9,10] proposed the compressed sensing in 2004. In recent years, the application of compression sensing in cardiac signal acquisition has gradually become a popular research direction among scholars, which has provided many research results. Hossein Mamaghanian et al. [11] applied compressed sensing to a multilead ECG acquisition system and proposed a method of combining compressed multilead ECG signals. Pawan K et al. [12] designed an ultra-low-power pulse oximeter sensor based on compression sensing, which is mainly used for long-term noninvasive monitoring of SpO_2_ and heart rate in Body Area Networks (BAN). Lee S et al. [13] proposed an ECG data compression algorithm based on K-singular value decomposition (K-SVD). The experimental results show that the algorithm has a high compression ratio and minimal data distortion. Zamani H et al. [14] used the block K-singular value decomposition (BK-SVD) algorithm to train a block sparse dictionary for PPG signals, and then used the block sparse Bayesian learning (BSBL) algorithm to recover the PPG signals using block sparse information. 

Biometric systems allow recognition and verification of an individual through his or her physiological or behavioral characteristics. It is a growing field of research due to the increasing demand for secure and trustworthy authentication systems. Bioidentification technology based on heart signals is an important research direction in the field of identification. Several studies have shown that cardiac-signal-based biometrics are feasible, and most research has been on biometric applications in its central electrical signal (ECG). Biel et al. [15] first proposed the feasibility of studying ECGs as biometrics. Hejazi M et al. [16] used kernel principal component analysis (KPCA) to extract the dimensionality features of ECG signals and classified them by support vector machine (SVM). Similarly, the literature shows the feasibility of PPG signals in the field of biometrics. Petros Spachos et al. [17] performed pulse (PPG) signal acquisition on the fingertips of 29 healthy subjects. The signals were preprocessed and projected into the LDA space, then the nearest-neighbor classifier was used for classification, verifying the feasibility of PPG as a biometric. A. Reit Kavsaolu et al. [18] used a second-derivative PPG (SDPPG) data card to collect 10 s of PPG signals from five healthy subjects and extracted 20 new features from each of them over a period of time. The features of the 20 PPG signals did not change, and the k-nearest-neighbor (K-NN) classifier was used to achieve a 95% recognition success rate with 10 new features of 10× cross-validation. Yathav et al. [19] developed a system called miBEAT that can simultaneously collect ECG and PPG signals and can be used on smartphones or tablets for signal acquisition and personal identification or authentication.

## 3. Heart Signal Processing Based on Compressed Sensing

### 3.1. Data Collection

This study focused on identification technology of cardiac signals based on compressed sensing, taking ECG and PPG signals in cardiac signals as the research objects. Acquisition of both cardiac signals was achieved via an AD8232-based [20] ECG acquisition module and a photoplethysmography module. Among them, the ECG acquisition module collected the chest ECG through lead I, and PPG signal acquisition was realized through the photoplethysmography sensor on the fingertip. In total, there were 23 subjects, including 15 males and 8 females, whose ages ranged between 23 and 25 years. A sampling frequency of 500 Hz was used.

### 3.2. Sparse Transform and Measurement Matrix Design

The sparseness of the signal was the prerequisite for the application of compression perception. The purpose of sparse signal representation is to concentrate the main information in the signal on as few nonzero coefficients as possible, simplifying the solution process. Commonly used sparse signal representation methods have different orthogonal basis functions and redundant dictionaries. In recent years, redundant dictionaries have been a research hot spot for the sparse decomposition of signals. There are two main ways to construct redundant dictionaries: (1) manual design and (2) dictionary training. Firstly, the construction method and sparse performance of the orthogonal basis function and redundant dictionary were studied. Discrete cosine transform (DCT) [21] and a K-SVD [22] redundant dictionary were used to determine the sparse representation method suitable for cardiac signals. The measurement matrix was chosen as the Gaussian measurement matrix, and to eliminate its randomness, the experimental results were averaged over the results of 10 runs.

**Figure 1:**
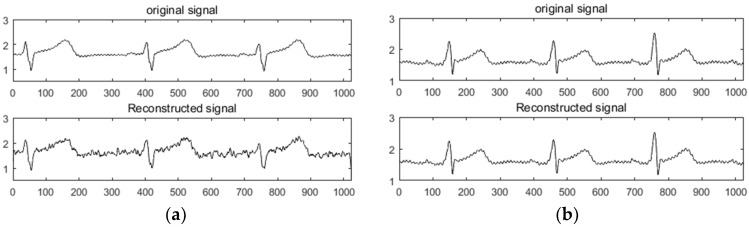
Reconstruction results of ECG under DCT + OMP and K-SVD + OMP. (**a**) DCT + OMP reconstruction results; (**b**) K-SVD + OMP reconstruction results.

**Figure 2:**
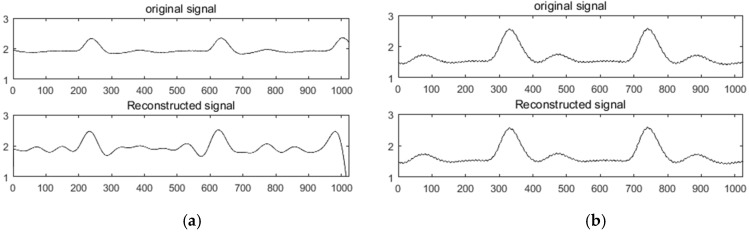
Reconstruction results of PPG under DCT + OMP and K-SVD + OMP. (**a**) DCT + OMP reconstruction results; (**b**) K-SVD + OMP reconstruction results.

The K-SVD redundant dictionary of ECG and PPG signals was constructed to simulate the experiment and compared with the DCT sparse basis. The collected ECG and PPG signals were used as experimental data. Firstly, 1,024,000 sampling points were collected as a sample set for dictionary construction, and the whole sample set was divided into atoms of length N (N=1024). The initial dictionary (dictionary D) was composed of K=800 atoms, and dictionary D was determined by the K-SVD algorithm. After training, the trained redundant dictionary D was obtained as an experimental dictionary. Then, the data containing at least 1024 sample points were collected as the original signal, the redundancy dictionary and the DCT sparse basis were used for sparseness, and the performance of the two sparse modes was analyzed and compared. In the comparison experiments of the two compression sensing methods, a random Gaussian measurement matrix and an orthogonal matching pursuit (OMP) reconstruction algorithm were used. For convenience of representation, the two methods were respectively recorded as DCT + OMP and K-SVD + OMP. As shown in Figure 1a,b, the experimental results of the ECG in the DCT + OMP and K-SVD + OMP compression reconstruction modes are shown in the compression ratio. Figure 2a,b shows the experimental results of PPG under DCT + OMP and K-SVD + OMP compression reconstruction modes in the compression ratio.

Figure 1 and Figure 2 show that the use of the DCT + OMP algorithm for ECG signal and pulse signal compression reconstruction resulted in serious distortion, while the use of the K-SVD + OMP algorithm for compression reconstruction of the two signals was significantly better than the former. Intuitively, the signal with less distortion represents the ideal reconstruction effect.

### 3.3. Refactoring Algorithm Design

In compressed sensing, the reconstruction quality of a signal is not only related to the selection of the measurement matrix but also to the reconstruction algorithm of the signal. A good reconstruction algorithm can more closely approximate the original signal and reduce the error before and after reconstruction. In this study, we mainly considered the SWOMP algorithm and improved it.

#### 3.3.1. Stagewise Weak Orthogonal Matching Pursuit Algorithm

The SWOMP algorithm [23] is an extension of the OMP algorithm, which selects the optimal atom for changing the index set according to the requirements of the inner product criterion metric. Unlike the OMP algorithm, only one maximum value is selected from the correlation vector at a time. SWOMP sets a threshold value when selecting an atom. Each iteration selects an atom greater than or equal to the threshold in the correlation vector and selects the corresponding atomic index. Incorporating atoms into the index set and support set improves the efficiency of the refactoring. In addition, SWOMP is also different from the stagewise orthogonal matching pursuit (StOMP) algorithm [24]. When selecting an atom, the selection of the threshold value is related to the residual coefficient by using the "weak selection" method, which reduces the requirement for the measurement matrix. Moreover, the OMP algorithm needs to input the sparsity *k* as a priori information, but the sparsity *k* value is often difficult to obtain. The SWOMP algorithm does not need the sparsity *k* value, and the signal can be reconstructed by setting the appropriate threshold and the number of iterations. Therefore, the SWOMP algorithm has a comparative advantage when reconstructing cardiac signals.

The reconstruction steps of the SWOMP algorithm are as follows.


**SWOMP algorithm:**
Input parameters: observation *y*, measurement matrix Φ, number of iterations S, threshold value α.Output parameters: sparse signal $\overset{\wedge }{\theta }$.Initialization: signal margin *r* = *y*, sparse signal $\overset{\wedge }{\theta }=0$, index set $\Lambda_{0}=\emptyset$, number of iterations k = 1, support set $A_{0}=\emptyset$.   (1) Calculate the correlation coefficient $u=\left\{u_{i}|u_{i}=|\langle r,\varphi_{i}\rangle |,i=1,2,\cdots ,N\right\}$.   (2) Select a value greater than $Th=\alpha \underset{i=1,2,\cdots ,N}{argmax}|\langle r,\varphi_{i}\rangle |$ in u and constitute the serial number corresponding to the selected value to form set $J_{0}$.   (3) Update the index set and the support set; let $\Lambda_{k}=\Lambda_{k-1}\cup^{\text{​}}J_{0}$ and $A_{k}=A_{k-1}\cup^{\text{​}}\varphi_{J_{0}}$. If $\Lambda_{k}=\Lambda_{k-1}$, stop iteration, and turn to Step (6).   (4) Calculate the least-squares solution of $y=A_{k}{\overset{\wedge }{\theta }}_{k},$
${\overset{\wedge }{\theta }}_{k}={\left(A_{k}^{T}A_{K}\right)}^{-1}A_{k}^{T}y$, and update the residual $r_{k}=y-A_{k}{\overset{\wedge }{\theta }}_{k}=y-{\left(A_{k}^{T}A_{k}\right)}^{-1}A_{k}^{T}y$.   (5) Let *k = k +* 1. If k≤S, return to Step (2); otherwise, stop iteration.   (6) Output sparse signal.

**Figure 3:**
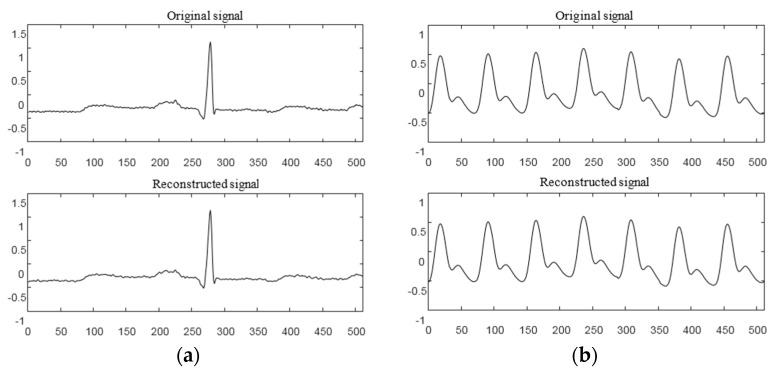
Signal reconstruction effect under the SWOMP algorithm. (**a**) ECG signal SWOMP reconstruction effect; (**b**) PPG signal SWOMP reconstruction effect.

Here, α has a value range of 0<α≤1 and the threshold Th=αmax{abs(u)}, $\phi_{J_{0}}$ represents the atom of the corresponding index set in the measurement matrix, and $\hat{\theta }$ is the reconstructed sparse signal. In order to verify the reconstruction performance of SWOMP, the redundant dictionary was selected as the sparse representation, the Gaussian random measurement matrix was used to observe the signal, and the SWOMP algorithm was used to reconstruct the signal. Figure 3a,b shows the reconstruction effects of the ECG and PPG signals under the SWOMP algorithm, respectively. The CSR of the ECG signal was 0.7 with a threshold value of α=0.83; the CSR of the PPG signal was 0.5, with a threshold value of α=0.7. It can be seen from the figure, before and after reconstruction, that the characteristics of the ECG and PPG signals were basically retained without obvious distortion.

However, the SWOMP algorithm also has some defects. The SWOMP algorithm selects the atomic update support set according to the established threshold value α, and the number of iterations *S* also needs to be artificially set. These two values are usually set empirically, which makes the SWOMP algorithm quite unstable. Because, usually, the choice of α value has a great influence on the reconstruction effect of the algorithm, if the value of α is too large (small), the number of selected atoms will be too small (more), which will result in poor signal reconstruction. Similarly, the selection of iterations number *S* also has an effect on the number of atoms selected. Moreover, the SWOMP algorithm selects multiple atoms larger than the threshold value at one time, unlike the OMP algorithm, which selects the best matching atom at one time. Although the reconstruction efficiency is improved and the requirement for the measurement matrix is reduced, the accuracy of reconstruction decreases when the selected atoms are not particularly suitable. So, the SWOMP algorithm needs further improvement in terms of atomic number selection.

#### 3.3.2. Improved Stagewise Weak Orthogonal Matching Pursuit Algorithm

First, two propositions are given:

Proposition 1: When the measurement matrix Φ satisfies the constraint equidistant property (RIP) with the parameter $\left(k,\delta_{k}\right)$, if $k_{0}\geq k$, then ${\left||\Phi_{\Lambda_{0}}^{T}Y|\right|}_{2}\geq \frac{1-\delta_{K}}{\sqrt{1+\delta_{K}}}{\left||Y|\right|}_{2}$ is the true proposition [25].

Proposition 2: The inverse of Proposition 1. When the measurement matrix Φ satisfies the constraint equidistant property (RIP) with the parameter $\left(k,\delta_{k}\right)$, if there is ${\left||\Phi_{\Lambda_{0}}^{T}Y|\right|}_{2}\leq \frac{1-\delta_{K}}{\sqrt{1+\delta_{K}}}{\left||Y|\right|}_{2}$, then $k_{0}\leq k$ is also a true proposition.

Here, $k_{0}$ is the estimated sparse value, *k* is the actual sparse value, $\Lambda_{0}$ is the index set corresponding to $k_{0}$ atoms, ${\left(\cdot \right)}^{T}$ is the transpose of (·), and $\Phi_{\Lambda_{0}}$ is the set of atoms in the corresponding index set $\Lambda_{0}$ in the measurement matrix.

According to Proposition 2, an initial sparsity estimate $k_{0}$ is obtained, and $k_{0}$ is selected as the initial minimum value of the atom. If the initial number of selected atoms is less than $k_{0}$, the modified threshold value α is reselected to the atom, ensuring the minimum value of the selected atom [26]. At the same time, combined with the idea of the sparsity adaptive matching pursuit (SAMP) algorithm [27], the algorithm can iterate adaptively according to the initial step size and initial residual. It is no longer necessary to set the iteration number *S* according to the empirical value. The iterative process is divided into several stages, and the phased selection of atoms in the support set is achieved according to the step size. As the iteration proceeds, the step size is updated and the support set is expanded. The iteration termination condition of the new algorithm is that the number of atoms in the support set will reach a certain number $S_{max}$. In order to prevent the number of atoms from being overselected, and to ensure the reconstruction accuracy, a number of reconstruction experiments were performed on ECG and PPG signals. From the experiments, it was found that the accuracy of reconstruction can be guaranteed when the maximum number of atoms does not exceed M/2. So, the maximum number of atoms was selected as M/2 in the improved algorithm and $S_{max}=M/2$ (*M* was the measured number). At the same time, after the atoms were selected at the threshold, the atoms were arranged in descending order to ensure that the most-matched atomic value was selected in the first place, so as to improve the reconstruction accuracy of the algorithm.

The steps of the improved sparsity weak adaptive matching pursuit (SWAMP) are as follows.

Input parameters: observation *y*, measurement matrix  Φ, threshold α, initial step size  L≠0, $\delta_{k}$.Output parameters: sparse signal $\overset{\wedge }{\theta }$.Initialization: signal margin r=y, sparsity $k_{0}=1$, sparse signal $\overset{\wedge }{\theta }=0$, index set $\Lambda_{0}=\emptyset$, $F_{0}=\emptyset$, support set $A_{0}=\emptyset$, maximum atomic number $S_{max}=M/2$.   (1) Calculate the correlation coefficient u by the formula $u=\left\{u_{i}|u_{i}=|\langle r,\varphi_{i}\rangle |,i=1,2,\cdots ,N\right\}$, and select an index corresponding to the $k_{0}$ maximum values from u to be stored in the index set $\Lambda_{0}$.   (2) If ${\left||\Phi_{\Lambda_{0}}^{T}Y|\right|}_{2}\leq \frac{1-\delta_{K}}{\sqrt{1+\delta_{K}}}{\left||Y|\right|}_{2}$, then $k_{0}=k_{0}+L$; turn to Step (1).   (3) Select T in $u=\left\{u_{i}|u_{i}=|\langle r,\varphi_{i}\rangle |,i=1,2,\cdots ,N\right\}$ that are greater than $Th=\alpha \underset{i=1,2,\cdots ,N}{argmax}|\langle r,\varphi_{i}\rangle |$. If $T<k_{0\;}$, decrease the threshold value α; turn to Step (3). If $T>S_{max}$, increase the threshold value α; turn to Step (3).   (4) Sort the selected atoms in Step (3) in descending order and select the first $k_{0}$ values. The corresponding serial numbers of the selected values constitute the set $J_{0}$, so $F_{0}=F_{0}\cup^{\text{​}}J_{0}$ and $\;A_{0}=A_{0}\cup^{\text{​}}\varphi_{J_{0}}$.   (5) Determine the initial residual $r=y-{\left(A_{0}^{T}A_{0}\right)}^{-1}A_{0}^{T}y\;$ for initialization stage stage=1.   (6) Select T values greater than $Th=\alpha \underset{i=1,2,\cdots ,N}{argmax}|\langle r,\varphi_{i}\rangle |$ in $u=\left\{u_{i}|u_{i}=|\langle r,\varphi_{i}\rangle |,i=1,2,\cdots ,N\right\}$, combine the sequence numbers corresponding to the selected values into a set $J_{t}$, and judge the selected atoms in descending order. If the number in set $J_{t}$ is less than or equal to the current step length *L*, turn to Step (7); if the number is greater than L, select the first L values and turn to Step (7).   (7) Update the index set and the support set, let $F_{t}=F_{t-1}\cup^{\text{​}}J_{0}\;$, and check the number in $F_{t}$. If it is greater than $S_{max}$, terminate; otherwise, $A_{t}=A_{t-1}\cup^{\text{​}}\varphi_{J_{t}}$.   (8) Calculate the least-squares solution of $y=A_{t}\overset{\wedge }{\theta },$
$\overset{\wedge }{\theta_{t}}={\left(A_{t}^{T}A_{t}\right)}^{-1}A_{t}^{T}y\;,$ and update the residuals $r_{t}=y-A_{t}{\overset{\wedge }{\theta }}_{t}=y-{\left(A_{t}^{T}A_{t}\right)}^{-1}A_{t}^{T}y$.   (9) Check the margin $r_{t}$. If $r_{t}\leq \varepsilon_{1}\left(\varepsilon_{1}\approx 10^{-3}\right)$, terminate; otherwise, stage=stage + 1, L=stage⋅L, and turn to Step (6);   (10) Output sparse signal $\overset{\wedge }{\theta }$.

**Figure 4:**
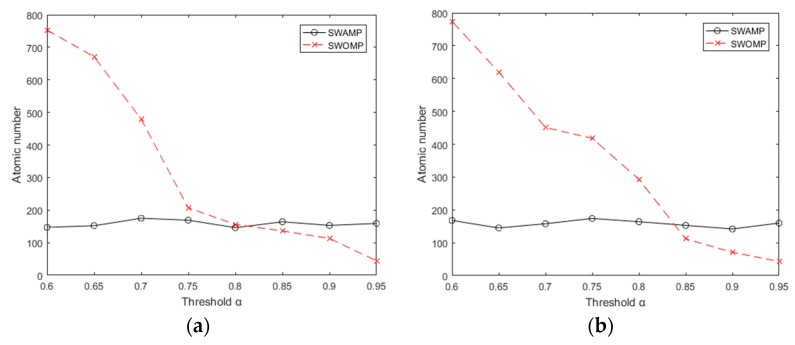
Number of atoms at different thresholds. (**a**) Number of atoms at different thresholds of ECG; (**b**) Number of atoms at different thresholds of PPG.

In order to more accurately compare the performance of these two reconstruction algorithms, a signal-to-noise ratio (SNR) was introduced as an evaluation index [28]. The SNR is defined in Equation (1):

(1) $$SNR=-20\log_{10}\frac{{\left||x-\hat{x}|\right|}_{2}}{{\left||x|\right|}_{2}}$$

where x is the original signal and $\hat{x}$ is the reconstructed signal. At the same time, the SWAMP and SWOMP algorithms were simulated and analyzed. The experiment followed the previous data, choosing the redundant dictionary as the sparse representation and the Gaussian random measurement matrix as the observation matrix. The range of the threshold was [0.60, 0.95], the compression ratio was 0.7, and the sparsity *k* was 100. To reduce the effect of the uncertainty of the random measurement matrix, the average was determined from 20 repetitions of the process. Figure 4a,b shows the number of atoms selected by the SWAMP and SWOMP algorithms under different threshold values. It can be seen from Figure 4a,b that when the threshold is small, the SWOMP algorithm excessively selects atoms; when the threshold is large, the SWOMP algorithm selects fewer atoms. Because the maximum expansion set of atomic selection is set, the atomic selection of the SWAMP algorithm is relatively stable, which is within the range of the maximum number of atoms specified by the algorithm.

**Figure 5:**
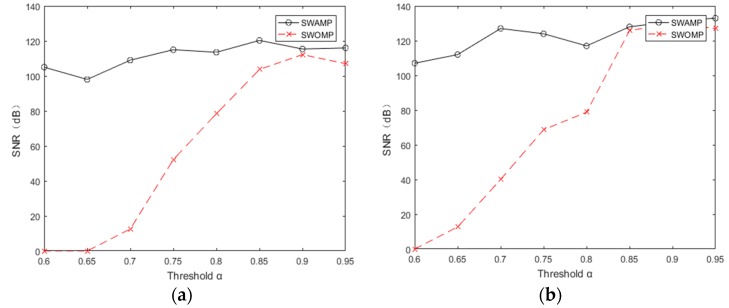
Signal to noise ratio at different thresholds. (**a**) SNR at different thresholds for ECG; (**b**) SNR at different thresholds for PPG.

Through the above analysis, we know that the choice of atomic number affects the reconstruction quality of the algorithm. Figure 5a,b shows the SNR of the algorithm at different threshold values. It can be seen from the figure that when the threshold values of ECG and pulse signals were taken as [0.65, 0.95], the SNR of the SWAMP algorithm did not change much and was stable within a certain range, and the reconstruction accuracy was higher than that of SWOMP. However, the SNR of SWOMP varied greatly, and only when the threshold value was greater than 0.85 did it have a higher SNR, indicating that the SWAMP algorithm has higher reconstruction accuracy and is more stable than the SWOMP algorithm.

In order to further verify the reconstruction performance of the SWAMP algorithm, the matching rate (MR) and root mean squared error (RMSE) were used to evaluate the quality of the reconstructed signal. The definition of the MR is shown in Equation (2) and the definition of the RMSE is shown in Equation (3):

(2) $$MR=1-\frac{{\left||x-\hat{x}|\right|}_{2}}{{\left||x|\right|}_{2}}$$

(3) $$RMSE=\sqrt{\frac{1}{N}\overset{N}{\underset{i=1}{\sum }}{\left(x\left(i\right)-\hat{x}\left(i\right)\right)}^{2}}$$

where x is the original signal and $\hat{x}$ is the reconstructed signal. Comparing the RMSE and MR values of the two algorithms under different compression ratios, the smaller the RMSE value, the larger the MR value, and the better the reconstruction performance of the algorithm. Table 1 and Table 2 show the reconstruction performances of the ECG and PPG signals under different compression ratios and compare them. It can be seen from the tables that, under the same compression ratio, the MR value of the SWAMP algorithm was greater than that of the SWOMP algorithm, and the RMSE value of the SWAMP algorithm was less than that of the SWOMP algorithm, indicating that the reconstruction effect of the SWAMP algorithm is better than that of the SWOMP algorithm, and that it has better reconstruction performance.

**Figure 6:**
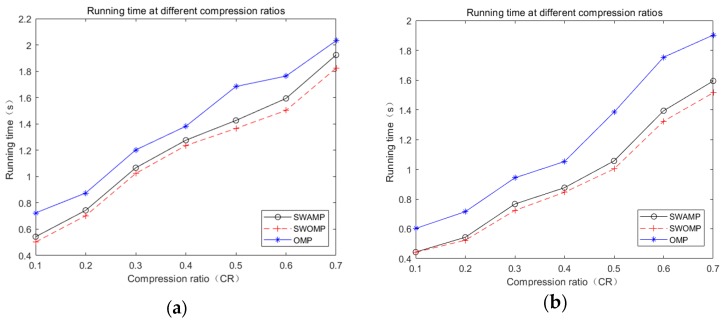
Run time under different algorithms. (**a**) ECG signals run time; (**b**) PPG signal run time.

The quality of an algorithm is also closely related to the reconstruction time. Reconstruction time is another indicator for judging the performance of a reconstruction algorithm. Figure 6a,b shows the ECG and pulse signal run times for the SWAMP, SWOMP, and OMP algorithms under different compression ratios.

It can be seen from Figure 6 that, because the threshold value was corrected and the minimum atomic selection value *k* was selected, the SWAMP algorithm’s run time was increased compared with the SWOMP algorithm, but the difference was not large. The run time was fast compared with the OMP algorithm, which ensured the reconstruction efficiency of the algorithm. At the same time, the SWAMP algorithm was more stable than the SWOMP algorithm, and the reconstruction accuracy was also improved. So, the SWAMP algorithm had better reconstruction performance.

## 4. Identification Based on Biological Electrical Signals

Human identification is essentially a pattern recognition problem involving three main steps: preprocessing, feature extraction, and classification. Preprocessing can be regarded as a noise and artifact removal step. Feature extraction operates directly on the Alternating Current (AC) of a few seconds of ECG to form distinctive personalized signatures for every subject. As in most pattern recognition problems, classification among a gallery set is the last step of the identification process. Based on the compressed sensing processing of ECG and PPG signals, this part preprocesses, extracts, and classifies the two signals separately and compares the feature extraction and recognition effects of the signals before and after reconstruction. 

### 4.1. Signal Noise and Pretreatment

Raw ECG data contain a considerable amount of noise that has to be eliminated. The most common types of noise in ECGs are baseline wander and powerline interference [29]. Baseline wander is caused by low-frequency components that force the signal to extend away from the isoelectric line. The source of this kind of artifact is respiration, body movement, or inadequate electrode attachment. Furthermore, powerline interference is generated by poor grounding or conflicts with nearby devices. Hejazi M [16] et al. denoised ECG signals by wavelet transform. Experiments have shown that wavelet transform based on the coif3 function has a better denoising effect. In this study, the two signals were preprocessed using the same method. First, preliminary denoising was performed by a high-pass filter hardware circuit, then denoising was performed by a wavelet transform algorithm based on the coif3 function. It (wavelet transform) can analyze the time domain and frequency domain of the signal at the same time.

### 4.2. Feature Extraction

In the process of heart signal recognition, it is necessary to locate and extract feature points and feature vectors after signal preprocessing, and the extracted feature vectors are used as the basis for recognition. Feature extraction of ECG signals is performed by means of discrete wavelet transform [30]. Wavelet transform can decompose the signal in the frequency and time domains by changing the frequency and position of a waveform, which satisfies the requirements of different resolutions in the time and frequency domains. The window size by wavelet transform varies—it is wide at low frequencies and narrow at high frequencies, and so is applicable to all frequency bands. An important application of wavelet analysis is its ability to process and compute data in compressed parameters, which are often referred to as features. The different frequency bands can be analyzed using discrete wavelet transform, where the signal is decomposed into an approximation and detail coefficients. The main advantages of the discrete wavelet transform are its robustness and flexibility for nonstationary ECG data analysis. The morphological characteristics of ECG signals have different peak amplitudes, peak intervals, and QRS complexes. The feature extraction method of wavelet transform was used to detect different peaks of ECG signals. After denoising the signal by wavelet transform, the corresponding threshold was set according to the characteristics of the transformed wavelet coefficients, so as to locate the QRS, P-wave, T-wave [31], as well as the starting and ending points of the P-wave and T-wave (denoted as PB, PE, TB, and TE).

**Figure 7:**
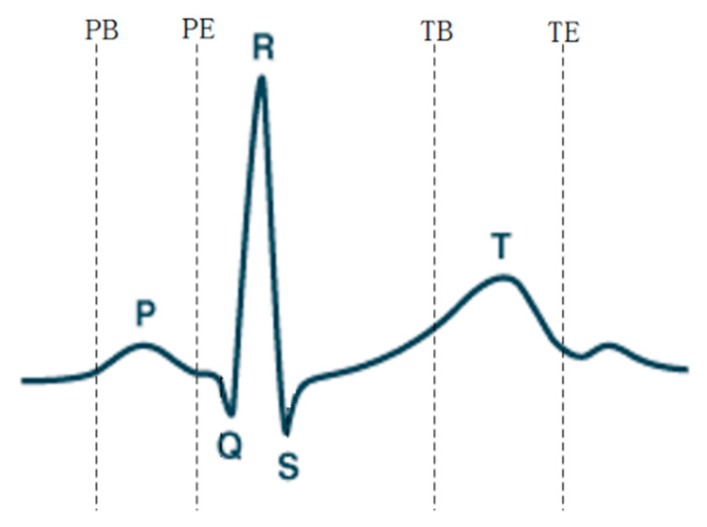
ECG signal reference point extraction.

**Figure 8:**
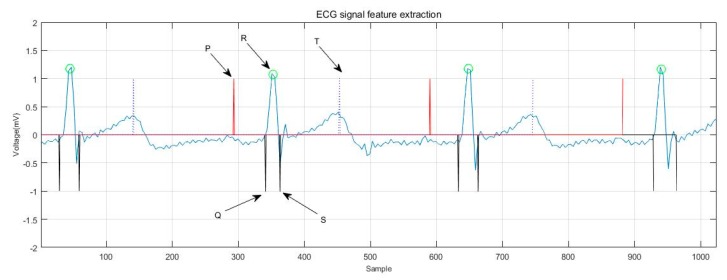
ECG signal feature extraction.

Figure 7 shows the extraction of ECG signal reference points. The distance and amplitude between each base point were calculated by numerical operation, and they were formed into eigenvectors. Independent classification of the subject’s heart rate required normalization of the 15 distance features using the average RR [17,32,33] distance of all subjects in the training group. The 15 range and six amplitude characteristics of ECGs are shown in Table 3. Figure 8 shows the ECG signal feature extraction diagram, in which the detected QRS band, PT, as well as the starting and ending positions of the PT band are annotated. 

**Figure 9:**
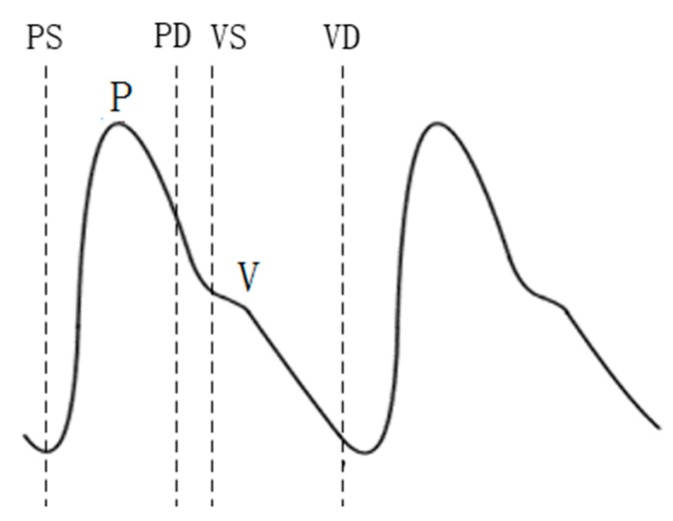
PPG signal reference point extraction.

**Figure 10:**
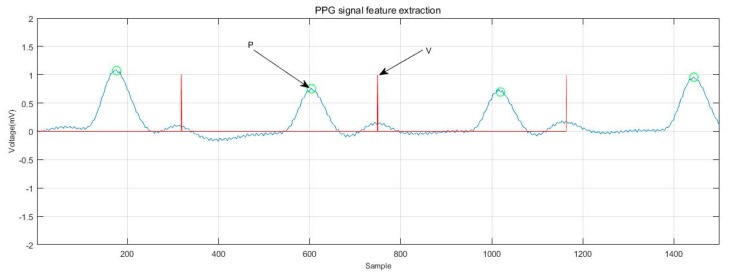
PPG signal feature extraction.

Compared with the extraction of feature points and feature vectors of ECGs, the extraction of reference points and feature vectors of PPG signals is relatively less common. At present, reference point extraction of PPG signals mostly adopts the signal derivative method [34,35]. In this study, the PPG signal was improved by means of wavelet transform to carry out feature extraction. The P-wave of the PPG signal was extracted, and the feature vector was selected as the reference point for recognition. The experiments showed that the method can accurately locate the reference point. The positions of the P and V waves of the PPG signal and their starting and ending points (denoted as PS, PD, VS, and VD) were detected. The extraction of the PPG signal reference point is shown in Figure 9. The normalization process was also performed before the recognition training, and the eight distance characteristics of the PPG and the four amplitude characteristics are shown in Table 3. Figure 10 shows the PPG feature extraction results.

### 4.3. Classifier Research

After extracting the features of signals, identification and classification were carried out. Because biological data is nonlinear, the SVM is considered to be the most suitable classifier among biometric classifiers based on ECG features [36]. At the same time, the SVM algorithm is suitable for being embedded into hardware modules [37,38], making the system compact and convenient. 

SVM is a binary classifier, and biometrics require multiple classifications. In this work, the one-to-one SVM [39] was used to classify the signals for identification purposes. The kernel function selected was the radial basis function (RBF), which is the most effective and commonly used method for ECG signal recognition with SVM [40]. The performance of an SVM classifier based on the RBF kernel function is generally affected by two parameters: penalty coefficient C, and RBF function parameter gamma [41]. The appropriate parameters were selected by cross-validating 10 times. In the training phase, the feature extracted from the test sample was input into an SVM classifier based on the RBF kernel function to obtain an SVM model, and an independent classifier was trained between each two individuals. In this study, there were 23 subjects, so 253 classifiers needed to be trained, and the most votes were used as the recognition results.

## 5. Algorithm Simulation Analysis

**Figure 11:**
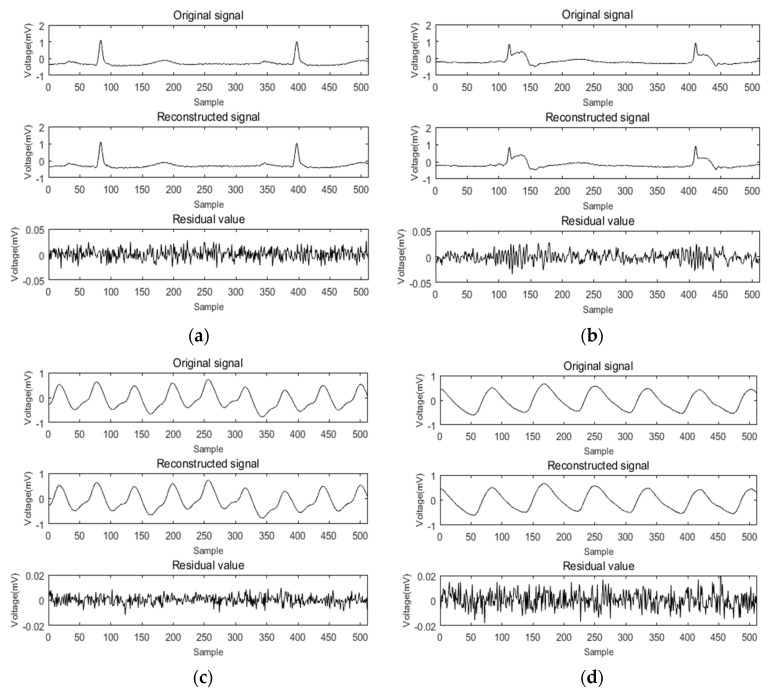
K-SVD + SWAMP reconstruction results. (**a**) ECG signal 101 reconstruction; (**b**) ECG signal 102 reconstruction; (**c**) PPG signal 112 reconstruction; (**d**) PPG signal 115 reconstruction.

ECG and PPG signal identification experiments were carried out using the methods described above. By comparing the feature extraction and recognition effect of the signal before and after reconstruction, the accuracy and practicability of compressed sensing technology applied to the identification of cardiac signals were verified. The collected ECG and PPG signals were used as experimental data. In the compression and reconstruction stage, at least 1,024,000 sample points were collected for each person as a sample set for dictionary construction, then the entire sample set was divided into atoms of length N. The initial dictionary (dictionary D) was composed of K = 800 atoms. Dictionary D was then trained by the K-SVD algorithm, and the trained redundant dictionary D was used as an experimental dictionary. Next, a signal containing 512 sampling points was randomly selected to be sparse through the trained redundant dictionary and was reconstructed using the random Gaussian measurement matrix and the SWAMP algorithm. The compression ratio was 0.7. For ease of presentation, the method was scored as K-SVD + SWAMP. The ECG signals of six of them were recorded as 101–106, and the pulse signals were recorded as 111–116. The reconstruction results of these 12 groups of signals were analyzed. The average SNR, MR, and RMSE values of the 12 sets of signal reconstructions are shown in Table 4. The average of the 10 reconstruction results was taken. Figure 11 shows a diagram of the reconstruction results of the ECG and PPG signals, respectively. The ECG signals numbered 101 and 102 were compressed and reconstructed, and the PPG signals numbered 112 and 115 were compressed and reconstructed.

Figure 11a,b shows the results of ECG signal reconstruction with numbers 101 and 102. It can be seen from the figure that there was no significant difference in the waveforms before and after reconstruction. The residual value was less than 0.05, the RMSE of 101 after reconstruction was 0.0086, and the matching degree was 97.26%. The RMSE of 102 was 0.0083 and the matching degree was 97.37%. Figure 11c,d shows the PPG signal reconstruction results of numbers 112 and 115, respectively. It can also be seen that the signal reconstruction was very similar before and after. The residual value was less than 0.02, the RMSE value of 112 after reconstruction of the ratio was 0.0016, and the matching degree was 99.56%. The RMSE of 115 was 0.0017, and the matching degree was 99.53%. The compression reconstruction method proposed in this paper had a good effect on the processing of bioelectrical signals.

It can be seen from Table 4 that the RMSE after reconstruction was kept at a small value, the SNR was large enough, and the error before and after signal reconstruction was small. However, the signal reconstruction effect was not the same. The analysis showed that the quality of the acquired signal had a certain influence on the reconstruction effect. The smaller the signal noise, the better the reconstruction effect. At the same time, the uncertainty of the random measurement matrix also had a certain influence on the experimental results. 

**Figure 12:**
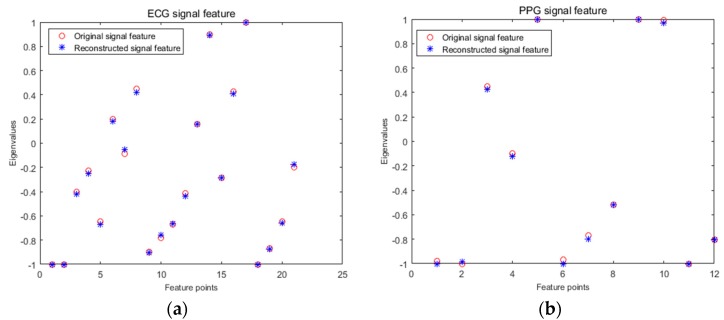
Comparison of features before and after signal reconstruction. (**a**) ECG signal feature; (**b**) PPG signal feature.

In the feature extraction and recognition stage, the practicability of compressed sensing in the identification of biological heart signals was analyzed by comparing the difference of the feature value and the change of the recognition rate before and after reconstruction. Firstly, a segment of the signal containing 1024 sampling points was selected as S1, and the S1 signal was compressed and reconstructed to obtain signal S2. Then, the S1 and S2 signals were extracted by wavelet transform, and the obtained eigenvalues were compared. A comparison between the ECG and PPG signals before and after reconstruction is shown in Figure 12a,b. ECG signals were extracted from a set of 21 features for the individual with recording number 101, and from a set of 12 features for the pulse signal with recording number 111.

From Figure 12, we can see that the proposed eigenvalues of the same signal before and after reconstruction basically coincided, indicating that the reconstructed signal basically retained the characteristics of the original signal, and the signal error before and after reconstruction was very small. The ECG signal before and after reconstruction and the feature values extracted by the PPG signal were used for classification and recognition. Three sets of features were extracted from each individual training sample via the method described above, and the data were normalized and sent to the SVM for training. The recognition samples were extracted from three groups of features for recognition, and the final output recognition results were 95.65% before and after ECG signal reconstruction and 91.31% before and after pulse signal reconstruction, with the same recognition rate before and after reconstruction. This indicated the effectiveness of the method proposed above for two kinds of cardiac signal compression reconstruction and the practicality of using it for cardiac signal classification and recognition.

## 6. Conclusions

In this work, we studied the compression and reconstruction methods of cardiac signals based on compressed sensing. The ECG and PPG signals in the heart signal were used as research signals. Firstly, the sparsity of ECG and PPG signals was studied, and the effects of ECG and PPG signals reconstructed using a single sparse basis and a redundant dictionary constructed by K-SVD were compared under the same measurement matrix and reconstruction algorithm. Obviously, the effect of the redundant dictionary of the K-SVD structure was better. Then, the performance of the SWOMP algorithm for the heart signal was analyzed. To address the disadvantages of the SWOMP algorithm (i.e., its lack of stability and low precision), an improved SWAMP algorithm was proposed. The simulation proved that the SWAMP algorithm is better than the SWOMP algorithm.

We also analyzed the application of compressed sensing in ECG and PPG signal recognition. Firstly, the ECG and PPG signals were predicted and processed by wavelet transform, and the feature of the reconstructed signal was extracted as the recognition basis by using the SVM. The experimental results showed that the recognition rate before and after reconstruction was the same, which further verified the accuracy and practicability of compressed sensing for portable remote heart signal identification.

## Figures and Tables

**Table 1:** Reconstruction performance of ECG signals via different reconstruction algorithms under different compression ratios.

Compression Ratio	SWAMP		SWOMP	
M/N	MR	RMSE	MR	RMSE
0.1	85.59%	0.0441	76.21%	0.0750
0.3	93.12%	0.0188	88.26%	0.0370
0.5	97.99%	0.0101	94.63%	0.0169
0.7	99.76%	0.0024	97.18%	0.0089

**Table 2:** Reconstruction performance of PPG signal by SWAMP algorithm under different compression ratios.

Compression Ratio	SWAMP		SWOMP	
M/N	MR	RMSE	MR	RMSE
0.1	88.44%	0.0156	74.76%	0.0796
0.3	95.36%	0.0064	91.58%	0.0265
0.5	98.68%	0.0032	98.13%	0.0067
0.7	99.89%	0.0011	99.31%	0.0025

**Table 3:** ECG and PPG signal features.

	ECG Signal Features	PPG Signal Features
Distance Feature	R-Q R-S R-P R-PB R-PE R-T R-TB R-TE PB-PE TB-TE Q-P S-T P-T Q-PB S-TE	P-PS P-PD P-V P-VS P-VD VS-VD PD-V PD-VD
Amplitude feature	Q-R S-R PB-P P-Q T-TB T-S	PS-P P-PD V-VS PD-V

**Table 4:** Average reconstruction performance of 12 groups of cardiac signals.

	Record Number	MR	SNR	RMSE
ECG	101	94.80%	69.14	0.0520
	102	98.51%	84.18	0.0149
	103	94.66%	85.38	0.0534
	104	97.16%	71.23	0.0284
	105	95.55%	72.25	0.0445
	106	92.94%	56.40	0.0706
PPG	111	92.44%	64.70	0.0702
	112	94.00%	56.28	0.0600
	113	93.72%	59.83	0.0628
	114	96.16%	65.18	0.0384
	115	97.12%	70.94	0.0288
	116	95.69%	62.89	0.0437
